# Exclusion and inclusion of parents of hospitalized children in Norway in the period 1877–2017

**DOI:** 10.1186/s12912-019-0330-6

**Published:** 2019-03-04

**Authors:** Hildegunn Sundal, Karin Anna Petersen, Jeanne Boge

**Affiliations:** 1 Department of Health and Social Care, Molde University College, PO. Box 2110, 6402 Molde, Norway; 2 Department of Global Public Health and Primary Care, University of Bergen, Faculty of Medicine Dentistry, Bergen, Norway; 3 Western Norway University of Applied Sciences, Bergen, Norway

**Keywords:** Children, Hospital, Parents, Exclusion, Inclusion, Michel Foucault, Discipline

## Abstract

**Background:**

Today, Norwegian parents have the right to stay with their children when they are in hospital. This right is relatively new. The purpose of this article is to examine the nursing profession’s ideas on how parents should be included/excluded when their children are in hospital, and to examine the social and ideological conditions that made the nursing profession’s ideas on inclusion/exclusion practices possible.

**Methods:**

The analyses are done in the tradition of the French philosopher Michel Foucault’s writings on how different kinds of knowledge have been used to discipline citizens. Such studies include analyses of descriptive and normative material and analyses of the ideological and social conditions that made the practices possible. The analyses are based on Norwegian textbooks on nursing.

**Results:**

Parents are rarely mentioned in Norwegian nursing textbooks from the period 1877–1940, and they are not present in photos from hospitals. The exclusion of parents may be due to the absence of welfare services and the fear of parents transmitting diseases from the hospitals to the general population.

The first Norwegian nursing textbook that argued for the importance of letting parents visit their children in hospital was published in 1941. In 1968, nursing textbooks started to argue for parents’ participation in the care. Since 1987, nursing textbooks have advocated full parental participation. The inclusion of parents was in accordance with humanistic ideology. The inclusion of parents occurred in a period of great nursing shortage. In this situation, it would have been of interest to entrust as much as possible of the nurse’s work to the family.

**Conclusions:**

Our conclusion is that ideas break through when they are in line with social conditions. From 1877 to 1940 social and economic conditions made it difficult for parents to be with their children in hospital, and hygiene ideology/theory contributed to legitimization of the exclusion of the parents in the care. During the period 1941–2017 it has been economically advantageous for the hospitals that parents care for their children. Ideas on the vulnerable child and self-help ideology have contributed to legitimization of the inclusion of the parents.

## Background

The purpose of this article is to examine the nursing profession’s ideas on how parents should be included/excluded when their children are in hospital, and to examine the social and ideological conditions that made the nursing profession’s ideas on inclusion/exclusion practices possible.

The background for the study are the stories the first author’s mother told about not being permitted to visit her children when they were in hospital in the 1950s to 1960s, in contrast to the first author’s experience of always being able to stay with her children when they were hospitalized between 1980 and 2000. The first author has long been interested in hospitalization of children, and has for years taught bachelor and master students about such practices. Her research is on the same topic.

Today, Norwegian parents have the right to stay with their children when they are in hospital. This right is relatively new. Documents show that parents in some periods were excluded from the care of their children in Norwegian hospitals [1–13]. National and international studies from the period 1986–2015 show that parents are included in the care of their children [14–22]. The previous studies do not explore the social or ideological conditions for the emergence of such exclusion and inclusion ideas and theories. This does, however, have a central place in Sundal’s [23] study on the exclusion and inclusion of parents when their children are in hospital.

## Methods

Analyses of Norwegian nursing textbooks had a central position in the Sundal [23] study and the analyses are done in the tradition of the French philosopher and historian of ideas, Michel Foucault’s, writings on discipline. Foucault argues that some forms of knowledge are used to promote discipline by means of regulations and devices, which control individuals from the outside, in harmony with what are considered socially useful practices. Objectifying knowledge has been used for external control of individuals. Foucault calls such control discipline. Other forms of knowledge are used for getting individuals to govern themselves. Subjectifying knowledge has been used to make individuals control themselves and in the context of this study this is called self-discipline [24, 25]. In line with Foucault’s thinking, this article is based on the assumption that during some periods the exclusion of parents from hospitals has been considered useful to society and during other periods the inclusion of parents has been considered useful, and different kinds of knowledge is used to support such ideas and theories.

As mentioned-above, Foucault’s expositions of discipline [regimentation] have served as analytical tools [24]. The study’s empirical object has thus been illuminated by an approach with corresponding archaeological and genealogical studies of the practices that emerge as inclusion/exclusion of parents when their children are in hospital [24–26]. Foucault has not explicitly described how he performed his studies. There is, however, some notes from a discussion between Foucault and French historians on how he examined discipline [regimentation] [26, 27]. In these notes, Foucault argues that studies of practices should include analyses of descriptive and normative material and analyses of the ideological and social conditions that made the practices possible. In the extension of Foucault’s methodological and analytical formulations, we have developed the following questions ([26, 27], p., 225):

The overall research question is: What has made it possible to think that parents should be included in the care of children in hospital at certain times and excluded at other times?

Research questions:

- *At what time did the nursing textbooks write about exclusion/inclusion of parents in the care?* [archeological approach]

b) *When did the parents become excluded/included in the care of hospitalized children?* [archeological approach]

c) *How were parents excluded/included in the care of their children in hospital?*
  [archeological approach].
d) *What was the ideological context that made parents’ exclusion/inclusion in care possible?* [genealogical approach]

e) *What was the social context that made parents’ exclusion/inclusion in care possible?* [genealogical approach]

### Empirical material

Different sources are used to answer the research questions a-e. In order to examine *at what time the nursing textbooks wrote about exclusion/inclusion of parents in the care*, Norwegian nursing textbooks were analyzed [i.e. Table 1].

**Table 1:** Norwegian textbooks on nursing

Nissen [2000] Textbook on Nursing. [reprinted from 1877]	
Waage [1901] Textbook on Nursing.	
Waage [1905] Textbook on Nursing. 2rd. ed.	
Waage [1911] Textbook on Nursing. 3rd. ed	
Grøn & Widerøe eds. [1921] Handbook on Nursing.	
Grøn & Widerøe eds. [1932] Textbook on Nursing. 2rd ed.	
Frølich [1921] Infant Care. In: Grøn, Widerøe, editors. Textbooks on Nursing.	
Frølich [1921] Infant Care. In: Grøn, Widerøe, editors. Textbooks on Nursing. 2rd ed.	
Jervell, Arentz, Asbjørnsen, Moe, Rimestad eds. [1941] Textbook for Nurses.	
Weberg, Sundal [1941] Care of the Healthy and Sick Infants. In: Jervell, Arentz, Asbjørnsen, Moe, Rimestad, editors. Textbook for Nurses Volume I.	
Jervell, Arentz, Asbjørnsen, Moe, Rimestad eds. [1951] Textbook for Nurses.	
Jervell, Wolan, Hunskaar, Thomassen, Nymoen, Thomassen eds. [1960] Textbook for Nurses. Volume I. General Nursing. 3rd ed.	
Jervell, Wolan, Hunskaar, Thomassen, Nymoen, Thomassen eds. [1960] Textbook for Nurses. Volume VI. Pediatric, Diseases and Infectious Diseases.	
Domaas, Heggenhougen, Wyller, Lindstøm, Vogt. [1951] General Care. In: Jervell, Arentz, Asbjørnsen, Moe, Rimestad editors. Textbook for Nurses. Volume I. 2rd ed.	
Hoven, Tveit, Tveit. [1960] General Nursing. In: Jervell, Wolan, Hunskaar, Thomassen, Nymoen, Thomassen editors. Textbook for Nurses. Volume I. 3rd ed.	
Lerheim, Borchgrevink, Breiland, Jukvam & Norwegian Nursing Association eds. [1968] Textbook for Nursing Schools. Volume III, Gynecology, Obstetrics and Pediatrics.	
Andersen [1968] Child Nursing. In: Lerheim, Borchgrevink, Breiland, Jukvam, Norwegian Nursing Association, editors. Textbook for Nursing Schools. Volume III, Gynecology, Obstetrics and Pediatrics.	
Tveiten [1987] Child Nursing.	
Grønseth & Markestad [2011] Pediatrics and Pediatric Nursing. 3rd ed.	

Such books are normative documents on how the nursing profession has thought practices should be, and we have examined how ideas and theories of exclusion and inclusion of parents have been embodied in such textbooks during the period 1877–2017. In this connection, we emphasized both what was written and what was not written about parents with children in hospital [28–45]. During the period 1877–1940 six Norwegian nursing textbooks were published with the same or different authors [28–35]. Between 1941 and 1986 three series of Norwegian nursing textbooks were published with the same or different authors [36–43]. All of these were included. During the last period, from 1987 to 2017, five separate Norwegian textbooks on pediatric nursing were published [44, 45]. All of these were included. Some editions and some volumes of the textbooks and series of textbooks on nursing and pediatric nursing are afterwards excluded, as they did not add anything new on parents in hospital [i.e. Table 1].

In order to examine *when the parents were excluded/included in the care of hospitalized children* we have analyzed photographs from Norwegian hospitals from all over the country [i.e. Table 2]. In those analyses we have assessed the situation pictured, what the situation is about, what the person[s] in these situations is [are] doing and who are present with the child/children etc. [2, 3, 7–9, 12, 13, 46].

**Table 2:** Photos of children and parents in Norwegian hospitals

Elster [1990] National Hospital.	
Ertresvaag [1993] Coastal Hospital in Hagevik through100 Years.	
Henry [1992] “I have the air, not to say scent, in nose yet.” In: Nord et al. editors. Coastal Hospital in Storm and Calm. Coastal Hospital at Stavern 1892–1992.	
Hustad [1993] Veranda Boy. In: Ertresvaag. Coastal Hospital in Hagevik through100 Years.	
Klepaker [1992] “Bad Memories throughout your Life.” In: Nord et al. editors. Coastal Hospital in Storm and Calm. Coastal Hospital at Stavern 1892–1992.	
Kvalvåg [1992] “Eternally Grateful that I was healed.” In: Nord et al. editors. Coastal Hospital in Storm and Calm. Coastal Hospital at Stavern 1892–1992.	
Seip [1993] A Historical Retrospective. In: Lie editor. For Sick Children in 100 Years. Pediatrics National Hospital, 1893–1993.	
Weium [2003] Marianne Hospitalized.	

In order to examine *how parents have been included/excluded*, we have analyzed Norwegian studies [17, 18], Norwegian articles [1, 46], a Norwegian novel [47], and Norwegian historic material on hospitals [2–6, 9, 10]. In our analysis of those texts we have emphasized both what was written and what was not written about parents with children in hospital [i.e. Table 3].

**Table 3:** Texts on children and parents in Norwegian hospitals

Elster [1990] National Hospital.	
Danielsen, Groven, Helgeland, Holte [2005] The Carers’ Experiences with Somatic Pediatric Wards in 2005 - Main Results of the National Survey. PasOpp Report: 03.	
Ertresvaag [1993] Coastal Hospital in Hagevik through100 Years.	
Evensmo [1954] The Glass Wall: Short Stories.	
Gade [1930] Coastal Hospital in Hagevik.	
Grindaker [1993] The Development of Pediatric Nursing. In: Lie, editor. For Sick Children through 100 years. Pediatrics National Hospital, 1893–1993.	
Lie [1993] editor. For Sick Children through 100 years. Pediatrics National Hospital, 1893–1993.	
Nord et al. eds. [1992] Coastal Hospital in Storm and Calm. Coastal Hospital at Stavern 1892–1992.	
Seip [1993] A Historical Retrospective. In: Lie editor. For Sick Children in 100 Years. Pediatrics National Hospital, 1893–1993.	
Sundal [1998] Children in hospitals. A Phenomenological Study of the Experiences Mothers have Admitted to Hospital together with their Children.	
Weium [2003] Marianne Hospitalized.	
Wergeland [1954] Children in Hospital.	

When we identified new ways of writing about parents in the above-mentioned text, and new ways of including/excluding parents in photos, we have examined *the ideological conditions that made those changes possible* (i.e. Table 4). Those analyses were based on Norwegian textbooks on nursing [28–30, 44, 45, 48–55], Norwegian health care history [56], Norwegian nursing history [57], Norwegian childhood history [58, 59], Norwegian historical books on hospitals [2–5, 7] and Norwegian other textbooks [60–62] [i.e. Table 4].

**Table 4:** Norwegian sources on ideological conditions

Aaser [1921] Hygiene. In: Grøn, Widerøe, editors. Textbooks on Nursing.	
Auestad, Killingmo, Nyhus, Pande [1971] When Children Need a Hospital. Mental Hygienic Aspects.	
Bowlby [1952] Maternal Care and Mental Health.	
Elster [1990] National Hospital.	
Ertresvaag [1993] Coastal Hospital in Hagevik through100 Years.	
Frønes [1998] The Norwegian Childhood. 2rd ed.	
Grindaker [1993] The Development of Pediatric Nursing. In: Lie, editor. For Sick Children through 100 years. Pediatrics National Hospital, 1893–1993.	
Grønseth & Markestad [2011] Pediatrics and Pediatric Nursing. 3rd ed.	
Hauen [1967] General Nursing Teaching. In: Lerheim, Borchgrevink, Breiland, Jukvam, Norwegian Nursing Association, editors. Textbook for Nursing Schools. Vol I.	
Henderson [1961] Norwegian Nursing Association. ICN. Nursing Fundamentals.	
Klepaker [1992] “Bad Memories throughout your Life.” In: Nord et al. editors. Coastal Hospital in Storm and Calm. Coastal Hospital at Stavern 1892–1992.	
Larsen [1921] General Nursing. In: Grøn, Widerøe editors. Textbooks on Nursing.	
Larsen [1932] General Nursing. In: Grøn, Widerøe editors. Textbooks on Nursing. Larsen A. General Nursing. 2rd ed.	
Lerheim, Borchgrevink, Breiland, Jukvam & Norwegian Nursing Association eds [1967] Textbook for Nursing Schools. General Nursing. Vol I.	
Lie ed. [1993] For Sick Children in 100 Years. Pediatrics National Hospital, 1893–1993.	
Lystad [1921] Eye, Ear and Nose Care. In: Grøn, Widerøe editors. Textbooks on Nursing.	
Lystad [1932] Eye, Ear and Nose Care. In: Grøn, Widerøe editors. Textbooks on Nursing. 2rd ed.	
Martinsen [1984] Nursing History. Audacious and not Timid Deaconesses. A Caring Profession Emerges from 1860 to 1905.	
Nissen [2000] Textbook on Nursing. [reprinted from 1877]	
Robertson [1970] Young Children in Hospital. [reprinted from 1958]	
Schiøtz [2003] People’s Health - the Country’s Strength from 1850 to 2003. Volume 2.	
Schrumpf [2007] Childhood History.	
Torp [1968] Pediatrics. In: Lerheim, Borchgrevink, Breiland, Jukvam, Norwegian Nursing Association, editors. Textbook for Nursing Schools. Volume III, Gynecology, Obstetrics and Pediatrics.	
Tveiten [1987] Child Nursing.	
Waage [1901] Textbook on Nursing.	
Waage [1905] Textbook on Nursing. 2rd ed.	
Waage [1911] Textbook of Nursing. 3rd ed.	

In order to understand the exclusion and inclusion of parents at different times, we also examined *the social conditions that made those practices possible*. These analyses were based on texts on Norwegian general history [63], Norwegian childhood history [59], Norwegian nursing history [64, 65], Norwegian health care history [56] and Norwegian article [66], [i.e. Table 5].

**Table 5:** Norwegian sources on social conditions

Lund [2012] Activity and Profession. Norwegian Nurses’ Association for 100 years [1912–2012]. Volume II.	
Martinsen [2003] Caring, Nursing and Medicine. History and Philosophy Essays.	
Nerbøvik [2011] Norwegian History1860–1914. 2rd ed.	
Schiøtz [2003] People’s Health - the Country’s Strength from 1850 to 2003. Volume 2.	
Schrumpf [2007] Childhood History.	
Utaker [2005] Michel Foucault about Liberalism and Neoliberalism.	

#### The search for texts and photos

Before the study started the first author had collected a considerable amount of material on parents when children are hospitalized, as she for years had been teaching Norwegian bachelor nursing students on the topic. She also searched BIBSYS [used at Norwegian libraries] and the databases Cinahl, Medline and ProQuest. Different combinations of the following keywords were used: Hospital, ward, nurse, children, parents, include, exclude, involve, participate, collaborate, cooperate, “children in hospital”, “parents of children in hospital”, “nursing to children in hospital”, “textbook on nursing”, “textbook on pediatric nursing”, “childhood history”, “history of Norway”, “nursing history”, “hospital history”, “health care history”. Literature that might contribute to answering the research questions were included. Generally, “surface reading” has been used to determine if the sources are relevant or not relevant to answer the research questions. This was done by reading the source’s title, table of contents and headings, introductions, summaries, conclusions and endings, as well as reading parts of the remaining text to reveal the contents. We also searched for photos in the same sources. Texts and photos that contributed to answering the research questions were included. During the reading process, keywords in the text were emphasized and noted in the margin as part of the analysis, and important photos and quotes were emphasized and collected. The first author collected the material.

## Results

Below we present the results from the analyses. The first part deals with the exclusion of parents. In the second part we address the inclusion of parents.

### Exclusion of parents

Below, we have argued that it is likely that the parents were excluded from the care of children in hospitals in the period 1877–1940, and we have accounted for ideological and social factors that made such exclusion possible.

#### 1877–1940: Parents are rarely mentioned in textbooks

Parents were rarely discussed in Norwegian nursing textbooks used during the period 1877–1940 [28–33], except that there seems to be some room for mothers to nurse their infants, “That the mother’s milk is the best and healthiest for the child is accepted (9, p. 134)”. It is pointed out that children should be fed with a feeding bottle if the mother cannot breastfeed the child [23, 28, 30–31, 34–35].

Just as parents had no central place in nursing textbooks during the period 1877–1940, they are also absent from sixteen available photographs of children hospitalized in the same period. In 1893 the National Hospital Children’s Department [Rikshospital] was established as Norway’s only specifically pediatric unit before 1950 [4, 9], and photographs from there suggest that parents were absent [2, 9, 23]. Also at photos from the Costal Hospital at Hagevik and at Stavern [Kysthospitalet i Hagevik og ved Stavern] [3, 7, 12, 13], specialized hospitals for treatment of tuberculosis [3, 6, 10], the parents are absent [3, 7, 12, 13]. A photo from the period 1934–1940 from The Coast Hospital at Stavern shows a 5 year-old boy visited by his mother and his siblings and suggests there is not a total ban on parental visits at that time [8].

#### Ideological conditions that supported parents’ exclusion

Health professionals’ interests in excluding parents and hierarchical parental discipline of children may have supported the exclusion. Knowledge about hygiene may also have supported exclusion practices, and there was no room for ideas of the vulnerable child that included the parents.

#### Health professionals’ interest in exclusion of parents

Professionally it may have been of interest to exclude parents. This may have made the children easier to handle for medical observation and treatment. Doctors had the opportunity to observe the disease and try out treatments by gathering the sick children in institutions with no outsiders looking in, and without interference from concerned parents. Doctors did, however, need assistants to help them with observations, treatment and care of the sick children, and towards the end of the 1800s, nursing as a skilled profession emerges. With the parents absent, these skilled nurses, along with unskilled caregivers, could take care of the children in hospitals [5, 24, 56, 57, 67].

#### Hierarchical discipline

The emergence of skilled nurses during the last half of the 1800s was probably a key condition for the exclusion of parents. At that time, there was a clear submissive hierarchy of doctors, nurses, unskilled nurses and hospitalized children and parents, and the modern nurses were expected to help make the children obedient and manageable observation and treatment. In order for medically interesting knowledge to be generated and practiced, such a hierarchy may have been an important factor [23, 24, 57].

In nursing textbooks published between1877–1940 children are mentioned when caring for them presents challenges, such as giving a child drugs with an unpleasant taste and smell. First, try to trick the child into taking the drug, and then use force if this does not succeed. Using cardboard to keep the child from using its arms and hands to rub its eyes at dripping and rinsing of the eyes is recommended. Getting someone else to help with this is also recommended. The parents are not mentioned [23, 28, 29, 31, 48–51].

Children as object for medical observation and treatment are evident in the photographs, and especially in one photo from National Hospital Children’s Department [Rikshospitalet] in 1910, where a young child is at the centre of a teaching situation with many doctors in attendance and no parents present [2]. Furthermore, a photo from the early 1900s from The Coast Hospital in Hagevik shows naked children lying on rows of beds and being given “light treatment”. Their eyes are covered and they lie in a prone position. Parents are not present [3, 23].

When children were hospitalized during the period 1877–1940, they could remain there for months, and they were subjected to careful observations and underwent harsh treatment. Under these circumstances, they had little or no opportunity to seek comfort and support from parents. Disobedience and lack of submission might lead to severe penalties [7, 23].

#### No room for the vulnerable child

Ideas and thoughts on immature and vulnerable children were central to the child survival movement and philanthropic movement that emerged towards the end of the 1800s [58]. The romantic ideas and thoughts about children and family arose in Norway and the rest of Scandinavia in the late 1800s. The ideas and reflections originated in the French philosopher Jean-Jacques Rousseau’s thinking, where the notion of the child’s intrinsic value has its beginnings [58, 59]. Ideas and thoughts about vulnerable children do not, however, seem to have had any central place in hospitals at the time, as that would have made it natural to include the parents in the care. Thoughts on the vulnerabilities of hospitalized children do not seem to have had any impact on Norwegian nursing textbooks during the period 1877–1940.

#### Knowledge about hygiene that supported exclusion practices

Ideas about miasmas and theories about microbes may have helped support the exclusion practices.

Miasmas are described as small particles that existed in water, soil and air. The contagions were considered fairly harmless, and infection spread from exhalations from soil, exhalations from breathing and from the skin to the sick person. The miasmas were only dangerous if they came into contact with excrement, disorder, garbage, animal exhalations and other filth. Key measures to strengthen resilience and immunity were adequate diet, clean clothes, removal of dirt and filth, a frugal lifestyle and social reforms. The first Norwegian health law from 1860 was steeped in a miasmatic understanding of health and disease [56].

The theory about microbes replaced the idea about miasma in the late 1800s. The breakthrough for microbe theory came when the German physician Robert Koch identified the tubercle bacillus. Antibiotics were not widely available until the mid 1900s, so regardless of whether the idea about miasma or the theory of microbes prevailed as an explanatory model for infectious diseases, the focus on light, ventilation, and strict hygiene, forced isolation and extensive reporting requirements were key in preventing the spread of diseases [56.

This is clearly expressed in Norwegian nursing textbooks used in Norway during the period 1877–1940 [23]. The texts give detailed descriptions of what causes dangerous exhalations in closed rooms with sick people and then describe how such exhalations may be prevented with ventilation, order, light, and cleanliness. “Such harmful, putrid air must be prevented or remedied. In order to ensure the greatest order and cleanliness, the air in a hospital room must be cleansed (28, p. 27)”. Furthermore, instruments and equipment that come into direct contact with the patient must be clean, as must hands and a whole range of ways to disinfect the surroundings is listed in the textbook of 1921, pointing out that disinfection is legally enforceable through the Health Act of 1860 [52].

In the textbooks from the period 1877–1940, the care of infants is emphasized, and cleanliness in infant nutrition has priority. The infant’s food must be clean, and this is best achieved through nursing the child. It is also important to bathe the baby or wash its skin and clean the baby’s navel and eyes to protect its health [30].

#### Social conditions for parents’ exclusion

Modern hospitals emerged in Western countries during the second half of the seventeenth century. At that time parents seem to be excluded from the hospitals. This time witnessed an extensive industrialization in Norway. Paid work increasingly moved out of the home [63]. The extended family was replaced by the nuclear family, and this little unit had to take care of jobs inside and outside of the home to feed themselves [59]. At this time, public welfare was underdeveloped in Norway, and if parents wanted to stay in hospital with their child, they would be dependent on financial help to maintain income and practical help to take care of the home [56]. Even though there was comprehensive industrialization in Norway from the late 1800s, transportation was limited, and this may have made parents’ visits to hospitalized children time-consuming.

Not only work was moved away from the home in connection with industrialization, the same happened to education. In pre-modern Norway, schooling and work training had mainly taken place within close proximity of the home. In the last half of the 1800s came the requirement that everyone should go to school, legalized by the Education Act of 1889 [59]. This meant that parents and children spent less time together than in the pre-modern society. One can also imagine that the transfer of training to teachers with accompanying separation between parent and child may have contributed to it being not so troubling to entrust children to professionals in hospitals. The above-mentioned ideas on miasmas and theories on microbes legitimated the exclusion of parents.

### Inclusion of parents

In the 1940s, nursing textbooks markedly change the ways they write about parents. This is when textbooks start arguing that parents should be with their children when they are in hospital. This change in argumentation arises during a period when antibiotics become widely available, there is a great shortage of nurses, the welfare state is established, and Henderson’s ideas about self-help have an impact on the nursing profession [23].

#### 1941–1967: Parents can visit the children

The first Norwegian nursing textbook to argue that parents should be able to visit hospitalized children was published in 1941 [36]. Such visits should, however, be limited due to the risk of infection [37, 44].

In textbooks published in the 1960s [39] the norms for visits are extended to visiting the child every day and possibly twice daily and the child’s emotional health is cited as reason for this. The fear of infections still give limitations in terms of glass walls between children and parents for the youngest children. Norms relating to the time of separation also seem to imply emphasis on emotional aspects of parents and children, and parents are given more time when leaving the child. The textbooks of 1951 [38] and 1960s [39] argued that hospitals should be reserved for patients who needed treatment and not simply care, and the fear of contagion is evidently reduced at this time. The availability of antibiotics may be the reason why the fear of infection has subsided during this period [40, 41].

The parents’ limited visiting opportunities are described in nursing textbooks from the 1950s [cfr. Above], and are confirmed by sources on Norway’s only children’s department until 1950. Arguments in defense of limited visiting time are based on the child’s best interest, risk of infections and personnel resources [5, 9]. This happens despite the change in public attitudes in favour of increasing parents’ visiting time that follow from the recommendations from the World Health Organization [WHO] international conference on children’s conditions in hospitals in Stockholm in 1954 [1]. This is further underlined by the fact that in order to combat children’s fear of hospitalization, Norwegian authorities in 1950 take the initiative to create an information film. Photographs taken in this connection show that parents are still absent in the pictured situations [46].

That parents have limited access to hospitals due to the risk of infection is also confirmed by a Norwegian short story from the 1950s [47]. The short story describes a meeting between a mother and her child through a glass wall when the mother comes to visit, a meeting where strong emotions unfold and the mother and her child cannot get close to each other [47]. The description is in line with other sources from the 1950s [1, 2, 5].

#### 1968–1986: Parents may care for the children

In 1968 a textbook is published [42] that argues for the extension of visiting hours. At this point the child’s need for parents is more important than the risk of contagion.

The textbook argues that parents should prepare the child before admission to hospital, be present in the receiving situation, medical examination included. Moreover, the book recommends that the nurse shows understanding for the situation and spends time on the separation between mother and child. The nurse should obtain information about the child when the child’s care is transferred from the parents, and parents should assist in the separation to protect the child. The book also recommends that parents get to comfort the baby when it is time to sleep and help the child with eating and personal hygiene. The parents shall also be invited to participate in the treatment of the child, and to sit with the child when it awakens from anesthesia. That parents can stay with their child in the hospital becomes an explicit option [43]. “Small children in hospitals should receive daily visits unless communicable diseases or special psychological factors contradict this (43, p. 305)”.

#### 1987–2017: Parents should participate in the care and treatment

In a textbook published in 1987, the parents are expected to participate in the care and treatment when children are in hospital. Such participation will help parents in understanding the importance of the child’s care and treatment. This means that the parents, in addition to caring for the child, should be present in situations of treatment, medical examinations and other procedures. In line with the arguments for the parents’ participation in the care of the child, the hospital shall facilitate such participation and also make sure the parents have a chance to leave the child for a short time with the knowledge that the child will be taken care of [44].

A textbook from 2011 considers it as given that parents should participate in the care of hospitalized children, the same way the parents do at home [45]. Parents wish to attend to the basic care of the child and comfort, support and be available for the child, in short, be parents. Meanwhile, the textbook points out that nurses and parents collaborate on the care and that the nurse assumes responsibility when needed. However, they return the responsibility for the care to the parents when considered appropriate. The textbook also emphasizes that parents should prepare the child and participate in any medical treatment and procedures, during which they should repeat important information to the child and help the child cope and process experiences associated with treatment and procedure situations. Moreover, it is important to reassure parents of the importance of their presence even when the child protests. There are still some limits to what is expected of parents in medical treatment and procedure situations, and the text emphasizes that parents may feel uncomfortable when entrusted with tasks they cannot handle. Among other things, parents should not be observing vital signs and should not have to hold the child in a difficult procedure situation, but should be there for their child. The textbook from 2011 is still in use in 2017 [45].

Textbooks from the period 1987–2017 argue for parents’ care of their children [cf. above], and other sources [ref. below] support the arguments in textbooks. A photograph from the National Hospital Children’s Department suggests that parents are present with their children in hospital. The photo is assumed to be from the period 1970–1990’s [2]. The presence of parents in the photos from hospitals is supported by national studies from the period 1998–2006 that indicate the parents’ inclusion in the care of hospitalized children. The studies also indicate that when parents have been included, their need for relief has not always been met [17, 18].

#### Humanistic knowledge that supported inclusion practices

The thinking about inclusion of parents when children are in hospital may have been supported by humanistic thinking or ideas on self-help and separation anxiety.

#### Self-help thinking and ideas

In 1967 the first Norwegian nursing textbook [53] is published that argue that patients should manage themselves and that they need to be self-reliant and independent [54]. This argument is clearly related to the American nurse Virginia Henderson’s need and self-help ideology.

Henderson argues that the individual needs to be self-reliant and that nursing should be performed in such a way that it contributes to the individual regaining independence as soon as possible [68]. Henderson’s self-help thinking is being continued by the American nurse Dorothea Orem. This is clearly expressed in the textbook from 1987 that specifically argues that parents are responsible for meeting the child’s needs because children are too immature to handle their own care [44]. In a textbook from 2011, which is still in use in 2017, Orem’s thinking is clearly present [45].

#### Thoughts and ideas about separation anxiety

The unfortunate consequences that children become “passive and silent” at separation from parents are pointed out in the textbook from 1968, arguing that the baby’s crying is a natural expression [55].

In the textbook from 1987 ideas or thinking about separation anxiety emerge more clearly, and the attachment and separation theory about the child by psychiatrist and psychoanalyst John Bowlby is central. The textbook also gives a detailed account of the psychoanalyst and social worker James Robertson’s description of the child’s “adaptation” through three separation stages when the child is separated from parents, and then redefine this process as grief phases [44].

The arguments in Norwegian nursing textbooks about parental inclusion emerge during the same period as John Bowlby’s ideas and thinking are recognized. The book Maternal Care and Mental Health by Bowlby [60] discusses the importance of attachment and separation and continuity of connection between child and mother. The thinking is known and recognized among professionals in Norway in the 1950s to 1970s. Bowlby describes the separation from the mother as causing separation reactions in the child [61]. The book Young Children in Hospital by James Robertson [1970] was available in a Norwegian translation in 1967 [62]. The child’s experience of the separation from her or his parents is described as the protest-, despair- and denial phase, and the mental hygiene principles for hospitalized children will best be served if the mother is admitted with the child. Robertson relies on Bowlby’s theory of attachment and separation [62].

#### Social conditions for parents’ inclusion

The thoughts on inclusion in textbooks in nursing emerge in parallel with the development of the Norwegian welfare state and in parallel with an expansive growth in healthcare and a related nursing shortage [64, 65]. The shortage of nurses must be seen in context with the strong growth of technological medicine, which came about when antibiotics came into general use. The shortage of nurses may have made it convenient to pass on as much as possible of the nurse’s work to family. To avoid the costs of several caregivers it may also have been of economical interest that parents perform as much of the care as possible when children are in hospital [56]. The LEON principle [lowest effective level of care] which was introduced to Norway in the 1970s is in harmony with the inclusion of parents. The political purpose behind the principle is to perform medical services in the cheapest possible way. In this connection, it could be argued that it is cheaper that parents take care of the children rather than paid caregivers. The extensive parental participation in our time is in accordance with the dominant economic ideology New Public Management [NPM], which prevails in Norwegian health care. NPM is based on the assumption that the public sector is too large and costly and that health care should be more effective. The ideology gained ground in the public sector in the 1990s [56, 66]. It is likely that parents’ participation in care can contribute to increased efficiency.

## Discussion

In this part of the text we have first discussed how parents became disciplined to not stay with the children during the period 1877–1940, and then discussed how the parents have been disciplined to stay with the children from 1941 until today. Then we discuss strengths, weaknesses and limitations of the study.

In this text, we have argued that parents were absent from Norwegian nursing textbooks used in the period 1877–1940 [28–33]. This absence is in line with photographs and other texts from the same period [2, 3, 7–9, 12, 13]. At the time parents were excluded, it would most likely have been financially difficult for large segments of the population to stay with their hospitalized children [56, 59, 63]. Objectifying ideas and theories about infection, and ideas about the separation of children and parents, contributed to the legitimizing of parental absence, and disciplined the parents to accept exclusion from hospitals [52, 55, 56, 60].

It was not until the mid 1900s that the French Enlightenment philosopher Jean-Jacques Rousseau’s thinking on vulnerable children became popular and the child’s best interest became a notion, although his ideas were known also at the time when parents were excluded from the care of children in hospital [58, 59]. However, at this time the social conditions were not ripe for the realization of such ideas or thoughts. After World War II parents’ participation in the care for hospitalized children has been considered economically beneficial to society at large, and during the period 1941–2017 parents have gradually been included in the care of their children when in hospital [36–45]. A great nursing shortage yields a situation where it becomes in the interest of the hospital to entrust as much as possible of the nurse’s work to the child’s family. This is also a period when antibiotics become widely available and the welfare state is established, and a time when Henderson’s ideas about needs and self-help had an impact on the nursing profession [58, 64–66]. Economic conditions may therefore have contributed to that the ideas of children’s vulnerability gained acceptance in the mid-1900s [55, 56, 60–62, 64–68]. Ideas do, in other words, break through when they are in line with economic conditions. Such links between humanistic ideologies and economic conditions may be referred to as subjectifying self-discipline [24, 25, 55, 56, 60–62, 64–68].

The limitations of this article are that its findings are based solely on normative literature on Norwegian nursing and on pictures and descriptive literature from Norway. However, although the study is based on Norwegian material, and although we have not directly observed how inclusion/exclusion practices take place in hospitals, our findings on exclusion/inclusion of parents in the care nevertheless seem to be representative of other parts of the Western world as well, as our study in many ways is in accordance with existing studies from Western countries on how parents are included/excluded [14–22]. The fact that pictures underscore the norms in the textbooks also contributes to the study’s reliability and credibility.

## Conclusions

The purpose of this article was to examine the nursing profession’s ideas on how parents should be included/excluded when their children are in hospital, and to examine the social and ideological conditions that made the nursing profession’s ideas on inclusion/exclusion practices possible. Our conclusion is that ideas break through when they are in line with social and economic conditions. From 1877 to 1940 the social and economic conditions made it difficult for parents to be with their children in the hospital, and hygiene ideology/theory contributed to legitimization of the exclusion of the parents in the care. During the period 1941–2017 it has been economically advantageous for the hospitals that parents care for their children when they are in hospital. Ideas on the vulnerable child and self-help ideology have contributed to legitimization of the inclusion of the parents in the care.

## Abbreviations

LEON principle: Lowest effective level of care
NPM: New Public Management
WHO: World Health Organization
